# Resource Construction and Evaluation for Indirect Opinion Mining of Drug Reviews

**DOI:** 10.1371/journal.pone.0124993

**Published:** 2015-05-11

**Authors:** Samira Noferesti, Mehrnoush Shamsfard

**Affiliations:** Faculty of Computer Science and Engineering, Shahid Beheshti University, Tehran, Iran; Hong Kong Baptist University, CHINA

## Abstract

Opinion mining is a well-known problem in natural language processing that has attracted increasing attention in recent years. Existing approaches are mainly limited to the identification of direct opinions and are mostly dedicated to explicit opinions. However, in some domains such as medical, the opinions about an entity are not usually expressed by opinion words directly, but they are expressed indirectly by describing the effect of that entity on other ones. Therefore, ignoring indirect opinions can lead to the loss of valuable information and noticeable decline in overall accuracy of opinion mining systems. In this paper, we first introduce the task of indirect opinion mining. Then, we present a novel approach to construct a knowledge base of indirect opinions, called OpinionKB, which aims to be a resource for automatically classifying people’s opinions about drugs. Using our approach, we have extracted 896 quadruples of indirect opinions at a precision of 88.08 percent. Furthermore, experiments on drug reviews demonstrate that our approach can achieve 85.25 percent precision in polarity detection task, and outperforms the state-of-the-art opinion mining methods. We also build a corpus of indirect opinions about drugs, which can be used as a basis for supervised indirect opinion mining. The proposed approach for corpus construction achieves the precision of 88.42 percent.

## Introduction

In recent years, the explosion of social media on the Web (e.g., blogs, review sites, discussion forums and social networks) provides a rich source of knowledge (e.g., facts and people’s opinions). In the past decade, there is a large body of literature on the fields of social media and public health to tackle the different tasks (e.g., providing a suitable representation of social networks [1]) and with diverse applications (e.g., side effects detection from user reviews [2] and evaluating the effectiveness of disease response strategies using networked metapopulation [3]).

In the medical sphere in particular, a lot of health-related user-generated content is now available on the Web, which shares information about the patients’ health conditions, diseases, and medicines they take, as well as outcomes and side-effects that they experience. Analyzing these opinions can be valuable for healthcare providers to provide better services, and for laypeople to be aware of others’ opinions and to benefit from their experiences to make informed decisions before using a service or product. However, the ever-increasing amount of opinions on the Web has raised the demand for developing automatic methods of analyzing and summarizing opinions. Opinion mining is the field of study that aims to automatically extract and classify people’s opinions towards entities.

Although opinion mining is usually associated with sentiment analysis, in some streams of research, there is difference between opinion mining and sentiment analysis [4]. Opinion mining aims at identifying opinions and attitudes in natural language texts while sentiment analysis intends to infer emotional states from the texts.

There are two types of opinions: direct and indirect [5]. Direct opinion is expressed directly on an entity or one of its aspects (e.g., “This drug is very good.”). In contrast, indirect opinion is expressed on an entity based on its effect on some other entities (e.g., “After taking this drug, my blood pressure rises.”). From another perspective, an opinion can be any one of the following two types: explicit and implicit [5]. An explicit opinion contains specific opinionated words or expressions. For example, the sentence “Accutane is amazing.” expresses an explicit positive opinion through the opinion word “amazing”. In contrast, an implicit opinion is an objective statement that implies an opinion due to the desirable or undesirable facts. The sentence “This drug decreased my vision”, has no explicit opinion word, but clearly implies a negative opinion about the drug, since this experience is an undesirable fact. Different kinds of opinions are described in section “Motivation and Contribution” with some examples.

Most of current studies carried out in the field of opinion mining have aimed at detecting direct opinions, especially explicit direct opinions. However, in some domains such as medical and economic, it is very common for users to express their opinions indirectly. In the drug domain, patients usually write about their experiences of drug effectiveness or side effects instead of expressing a direct opinion. Patients’ experiences are often expressed without any explicit expression of opinion. Rather, the desirable or undesirable effects of the drug implicitly indicate a positive or negative sentiment towards the drug.

Identifying indirect opinions is very challenging but critical for facilitating implicit opinion mining and improving the overall performance of opinion mining systems. Therefore, in the current research, we focus on the task of indirect opinion mining. In this context, the aim of this paper is to: (1) propose a model for representing an indirect opinion; (2) construct a knowledge base of indirect opinions from drug reviews that can be used as a basis for the task of automatic indirect opinion mining; (3) build a corpus of indirect opinions which can be used as a resource for supervised indirect opinion mining; (4) evaluate the quality of the constructed resources and demonstrate the appropriateness of the proposed approach; and (5) propose and validate a method for polarity detection of new examples of drug reviews by employing the constructed knowledge base.

The rest of this paper is organized as follows. First, we provide an overview of related research in the area of opinion mining. Then we present the contribution of current work and some motivating examples to illustrate how the proposed approach improves existing ones. After that, we propose a model for representing indirect opinions. We present the exploited resources and the details of the proposed method for automatically constructing a corpus and a knowledge base of indirect opinions. We then report experimental results. The last section concludes the paper and outlines future directions.

## Related Work

Opinion mining has been a very active research area in recent years and, hence, there is a large body of research literature on this filed. Pang and Lee [6], Liu [5] and Cambria and Hussain [7] have provided comprehensive surveys of important research in this field.

Opinion mining contains many research areas. In this section, we just focus on two relevant research areas: (1) polarity classification which aims to classify texts into different categories (usually, positive or negative), and (2) resource creation for opinion mining. In the following subsections, we first review related work on polarity classification task. Then, we provide an overview of existing approaches to resource creation for opinion mining.

### Polarity Classification

We categorize existing work on polarity classification according to several dimensions: type of text, level of analysis, type of opinion, and technique that is used for analysis.

The first dimension is the type of texts on which the analysis is done. An opinionated text can be a review, an article in a newspaper, a forum discussion, a blog, a posting in a social network site, etc. Each of these types has its own special characteristics. For example, reviews are often more subjective than newspaper articles. Furthermore, analysis of reviews is more challenging than that of newspaper articles due to the informal style of writing and misspelling errors. Therefore, polarity classification requires different analysis techniques depending on the type of text.

Among these types of text, reviews have received more and more attention. Nowadays, individuals and organizations are increasingly using the content in review sites for decision making. Most of works on review mining have been carried out on general domains such as product, movie, hotel and restaurant reviews [8,9,10]. We aim at working on medical domain because of significant amount of indirect opinions and publicly available domain knowledge in this field. In addition, health-related social media offers a rich source of patients’ opinions. Analyzing these opinions could lead to an improvement in health-care services.

Second dimension is the level of analysis. Polarity classification has been studied at the document, sentence and aspect levels. The pioneered works focused on the document level aiming at determining the overall polarity of a document [9,11]. Document-level opinion mining is less effective since it assumes that each document is about a single object. However, this assumption is not always true.

Later works applied opinion mining to individual sentences in a document. Sentence-level opinion mining is associated with two tasks; the first task is to identify whether a given sentence is opinionated or not, called subjectivity classification [12,13], and the second one is to classify a sentence as a positive or negative opinion, called polarity classification [8,9,14,15].

Neither document-level nor sentence-level polarity classification is able to discover what exactly an author likes or dislikes. Thus, a more fine-grained analysis is needed. The task of aspect-level polarity classification determines the opinions expressed on different aspects of entities [8,16,17,18,19].

Third dimension is the type of opinion. Previous studies on opinion mining have mainly focused on direct opinions, especially on explicit direct opinions. Few studies have also been carried out on analyzing implicit opinions. Zhang and Liu [20] focused on the objective nouns and noun phrases that imply opinions. In a slightly different direction, Greene and Resnik [21] studied the influence of syntactic packaging on implicit sentiments. Cambria and Hussain [7] introduced a new paradigm, namely “sentic computing”, for concept-level opinion mining, which uses affective ontologies and common sense reasoning tools and is useful for inferring implicit emotional meaning underpinning words. However, none of these approaches has clearly focused on indirect opinions.

Fourth dimension is the technique used to tackle the task of polarity classification. Generally, existing approaches for opinion mining can be classified into three main groups: (1) Lexicon-based approaches [16,22], which mainly focus on the construction and use of sentiment lexicons such as SentiWordNet [23]; (2) Machine learning approaches [9,24,25], which depend upon the availability of an annotated corpus with polarity labels to train a classifier; and (3) Concept-based approaches [4,26,27,28,29], that focus on semantic analysis of text through the use of ontologies or semantic networks, which allow the aggregation of conceptual and affective information associated with natural language opinions.

As mentioned before, previous approaches have mainly focused on direct opinions and are not able to handle indirect opinions effectively. Lexicon-based and machine learning approaches mainly rely on the subjective part of text in which opinions are expressed explicitly, e.g., opinion words and their co-occurrence frequencies, while most of indirect opinions have no explicit opinion word. Even when machine learning approaches are employed to learn implicit opinions from corpora, the performance is low. The main reason is that they are semantically weak, meaning that with the exception of obvious opinion words they have little predictive value individually [7]. Concept-level approaches step away from blind use of keywords and word co-occurrence count, but rather they rely on the implicit features associated with natural language concepts and, hence, are able to handle some of implicit expressions of opinions. The focus of current concept-based approaches, however, is more on developing and/or employing a knowledge base of common sense concepts with associated polarity such as SenticNet (*http://sentic.net/senticnet-2.1.zip*). The accuracy of these approaches is dependent on the richness of the knowledge bases. However, due to the fact that these knowledge bases often do not contain technical and domain terms which occur frequently in the drug domain, they are insufficient for polarity classification of drug reviews.

Moreover, relying on the common sense and domain terms alone is not sufficient for predicting the polarity of indirect opinions since most medical terms such as “pain”, “depression” and “anxiety” are considered negative in current common sense knowledge bases such as SenticNet, but they occur frequently in positive sentences. For example, the sentence “After about 5 weeks, my acne disappeared and has not come back.” is positive although the concept “acne” is negative in SenticNet. In fact, verbs play an important role in analyzing the indirect opinions’ polarity. Thus, we need a different analysis technique for indirect opinion mining that not only spot domain terms but also considers context words such as verbs.

### Resource Creation for Opinion Mining

Opinion mining has many subtasks and for each of them, appropriate resources have been created. Therefore, there is a wide variety of researches on resource creation for opinion mining. In the rest of this subsection we just provide a review of some relevant studies conducted on creation of corpora, lexicons and knowledge bases for polarity classification.

Availability of labeled data is a prerequisite for development of supervised machine learning approaches. Therefore, many attempts have been done on developing a manually annotated corpus for opinion mining [8,16,30,31]. In [31] the main issues related to the development of a corpus for opinion mining were discussed. However, manual annotation of sufficient data is a tedious, expensive and time consuming task. In order to overcome this problem, researchers have proposed automatic approaches for corpus construction. Kaji and Kitsuregawa [32] proposed some heuristics to develop a polarity-tagged corpus from HTML documents based on Web page layout structures and linguistic patterns. In [33,34] Twitter was used to form a training set by using emoticons such as “:-)” and “:-(“. Asmi and Ishaya [35] proposed a framework for automatic generation of a corpus based on semantic analysis of text using existing resources (i.e., SentiWordNet, WordNet and domain specific dictionaries). The focus of current approaches has been on annotating direct opinions. Thus, existing corpora are not appropriate for indirect opinion mining. To the best of our knowledge, there is no previous work on automated building a corpus of indirect opinions.

In the context of lexicon construction, Turney and Littman [36] tried to find the semantic orientation of a word through the strength of its association with a set of seed words with known polarity. Esuli and Sebastiani [23] developed a lexicon called SentiWordNet employing eight classifiers and quantitatively analyzing the glasses of WordNet synsets to attach polarity values to each synset. Neviarouskaya, Prendinger and Ishizuka [22] generated SentiFul, a reliable lexicon of sentiment-conveying terms, modifiers, functional words and modal operators. However, there is no general-purpose sentiment lexicon since the polarity of words is domain-dependent. Hence, some of previous studies have focused on adapting sentiment lexicons to a specific domain [8,37,38]. However, same words in a same domain may indicate different polarities. In other words, sentiment polarity of words is context-dependent. To solve this problem, construction of context-dependent sentiment lexicons has been proposed [10,19]. In this kind of lexicons, polarity of a word is determined depending on its aspect in the context. Current sentiment lexicons are not appropriate for indirect opinion mining since most of indirect opinions are implicit and have no opinion words.

Some studies have also been carried out on construction of concept-level resources for opinion mining [4,26,27]. SenticNet is a resource for opinion mining that exploits AI and semantic Web techniques to attach polarity values and strength to common sense concepts. In [39] a new approach is represented for building a sentiment dictionary using iterative regression and a random walk strategy. Current concept-level resources are not sufficient for indirect opinion mining since the polarity of an indirect opinion is not only conveyed by its concepts but also by interaction of the concepts. However, previous approaches have considered concepts separately and not integrated their interaction.

A considerable number of studies have also been conducted on resource creation in closely related fields such as emotion detection. In [40,41] a method based on commonsense knowledge is proposed to the task of emotion detection which solves the problem of indirectly mentioning of an emotion. In this work, a knowledge base is built for modeling affective reactions to real-life situations described in text based on the appraisal theories. However, in practice, there are some limitations to using this method for indirect opinion mining of drug reviews. First, in this method, the initial core of the knowledge base was designed manually, and then following a semi-automatic process, it was extended and populated using real examples from the ISEAR data bank [42]. In fact, this method relies on labeled examples which may be not available in other domains. Second, this method is a commonsense knowledge-based approach while most terms of drug reviews are technical. Third, this method could not satisfactorily deal with the missing entity issue that occurs frequently in drug reviews. In summary, this method is a commonsense knowledge-based approach relying on labeled examples to the task of emotion detection. In other words, in order to be applicable in other domains or other tasks, this method needs appropriate changes and domain-specific labeled examples.

In this paper we aim to introduce a full-automatic approach which provides the essential resources for mining indirect opinions. The proposed approach does not need labeled examples and, hence, is applicable to any domain in which related domain knowledge is available.

## Motivation and Contribution

As mentioned before, an opinion can be one of the following two types: direct and indirect [5]. From another point of view, opinions can be grouped into two categories: explicit and implicit [5]. Table 1 shows different kinds of opinions according to different perspectives with some examples. For example, in Table 1 direct reviews (1–3) are explicit since the words “effective” and “amazing” imply positive sentiments and the word “dangerous” implies a negative sentiment. Likewise, in indirect reviews (6–7), the words “successfully” and “cure” imply positive sentiments. In contrast, direct reviews (4–5) and indirect reviews (8–12) are implicit since they do not have any opinion word.

**Table 1:** Different kinds of opinions with some examples

Type	Review
direct/explicit	(1) The drug was very effective.
direct/explicit	(2) This drug was amazing.
direct/explicit	(3) This drug is dangerous.
direct/implicit	(4) This drug is piece of crap.
direct/implicit	(5) This drug should be taken off the market.
indirect/explicit	(6) Taken daily, Prevacid successfully reduced my acid stomach symptoms to the point that they did not occur.
indirect/explicit	(7) It cured the acne.
indirect/implicit	(8) This drug reduced my pain significantly.
indirect/implicit	(9) This drug decreased my vision.
indirect/implicit	(10) This may lead to decreased renal function.
indirect/implicit	(11) It raised my LDL cholesterol level.
indirect/implicit	(12) After using this drug, my blood pressure rises.

Our studies on drug reviews from *www.druglib.com* show that only 27 percent of opinionated sentences actually contain direct opinions. In other words, about 73 percent of opinionated sentences are indirect. Only 48 percent of indirect opinions are explicit, which means that traditional approaches, which rely on subjective statements, only consider portions of the available data and ignore a considerable amount of valuable information. This is why exploring a model to deal with indirect opinions has been planned in the present research to solve the problem to a great extent.

To introduce the motivation behind our proposed approach and to illustrate in what way it improves the existing ones, we use some examples. Some opinions contain opinion words, and hence can be classified using traditional approaches. Given a sentence such as review (1) in Table 1, a lexicon-based system would be able to correctly classify it as positive since the opinion word “effective” has positive polarity in sentiment lexicons such as SentiWordNet. This is the case for reviews 2, 3, 6 and 7 as well. However, in review (8), although the negative word “pain” is present, the sentence should be classified as positive. To overcome this issue, some previous approaches defined additional rules. Liu [5] introduced a set of rules for dealing with sentiment shifters. One of these rules expresses that decreasing or increasing the quantity associated with an opinionated item can change the polarity of the opinion. However, this rule is only applicable for the sentences with opinionated items. Let us consider a more complicated example. Review (9), cannot be correctly classified by lexicon-based approaches or even by the additional rules which defined in [5] since it has no opinion word. A method to overcome this issue is sentic computing [7], whose main idea is to acquiring the polarity of different concepts based on commonsense knowledge. To correctly classify an indirect opinion such as review (9), the system should know that the expression “decrease vision” is a commonsense concept that produces a negative polarity. However, some contexts such as reviews (10–12), in which most of the concepts are technical, cannot be correctly classified by commonsense knowledge-based approaches. In fact, analysis of indirect opinions needs deep understanding of textual context, drawing on both commonsense and domain knowledge as well as linguistic knowledge.

To address the mentioned considerations, this paper explores the task of indirect opinion mining from unlabeled free-format textual user reviews. Analysis of indirect opinions is very challenging for several reasons. Firstly, as mentioned before, most of indirect opinions are implicit so polarity detection of them needs semantic analysis of text. Secondly, polarity analysis of indirect opinions requires domain knowledge since they often contain technical concepts. Thirdly, due to the informal writing style of user reviews, most of indirect opinions have a missing entity. For example, the sentence “reduced amount of cystic acne.” expresses a positive opinion about “Accutane”. However, the drug name has not been mentioned in the sentence. We term this as missing entity issue. In this paper, we propose a new model to deal with these challenges. The main contributions of this paper can be summarized as follows:

1. We propose a model for representing indirect opinions. In contrast to direct opinion mining for which there has been a great research, the problem of indirect opinion mining is almost unexplored.
2. We propose a novel semantic-based method to construct the essential resources for indirect opinion mining. Different from previous concept-level approaches for opinion mining, which mainly depend on commonsense knowledge, the proposed method relies on domain knowledge instead. In addition, the proposed method is fully automatic and does not require labeled examples.
3. We explore the special issues associated with indirect opinion mining, i.e., determining the possible types of entities which are talked about in a review site (section “Named Entity Recognition”), missing entity issue (section “Incomplete Indirect Opinion Extraction”) and missing knowledge issue (section “The proposed Method for Exploiting the OpinionKB”), and present a model based on domain knowledge aiming to mitigate these issues. To the best of our knowledge, these issues have not been addressed by existing approaches in the field of indirect opinion mining.

## Problem Definition

Before solving any scientific problem, we need to formalize it. In the following, we present indirect opinion mining formulation including the definitions, sub-tasks and objectives.

An indirect opinion is expressed on an entity, called “effective entity”, or an aspect of this entity based on its effects on another entity, called “affected entity”. Consider the following sentences:

e.1) “Yasmin is supposed to alleviate PMS symptoms.”

e.2) “A Yasmin overdose may cause bleeding.”

The above sentences consist of the following key components: (1) effective entity or opinion target that is an entity (e.g., “Yasmin” in e.1) or aspect of the entity (e.g., “overdose” of “Yasmin” in e.2) about which the opinion is expressed; (2) affected entity on which the effect of the effective entity is expressed (i.e., “PMS symptoms” and “bleeding” in e.1 and e.2, respectively); (3) effect which expresses the relationship between effective and affected entities (i.e., “alleviate” and “may cause”, respectively); and (4) opinion polarity which indicates whether the opinion is negative or positive. In the above examples, the first sentence has positive and the second sentence has negative polarity. Given this insight, we define an indirect opinion as follows.

**Definition 1** (indirect opinion): An indirect opinion is represented by a quadruple (*e*_*i*_, *e*_*j*_, *r*_*ij*_, *p*); where, *e*_*i*_ is the name of the effective entity, *e*_*j*_ is the name of the affected entity, *r*_*ij*_ is the effect of *e*_*i*_ on *e*_*j*_, and *p* is the opinion polarity. Depending on application, this definition could be extended by other components such as aspect of the effective entity on which the opinion is expressed, opinion holder and opinion time. In this research, we only consider four mentioned components.

**Definition 2** (indirect opinion mining): Considering the above definition, indirect opinion mining aims to discover all quadruples (*e*_*i*_, *e*_*j*_, *r*_*ij*_, *p*) in a set of opinionated texts *d*.

The sub-tasks of the defined problem are derived from the components of the mentioned quadruple. Some of them are similar to the sub-tasks of the direct opinion mining [5].

**Definition 3** (entity extraction and classification): This task aims to extract effective and affected entities, and categorize synonymous entity expressions.

**Definition 4** (relationship extraction): This task aims to extract the relationship between effective and affected entities. Thus, it is similar to the relation extraction task in information extraction [43].

**Definition 5** (polarity detection): This task determines the polarity of an indirect opinion.

## Methods and Materials

In this section, a novel approach is proposed to automatically build a polarity-tagged corpus and a knowledge base of indirect opinions as two resources for polarity detection of drug reviews. In the following subsections, we first present the datasets and resources used for this purpose. Then, we describe our proposed method for resource construction in detail. Finally, the proposed method for exploiting the constructed knowledge base for the polarity detection task is presented.

### The Exploited Resources

In order to construct the essential resources for indirect opinion mining, a dataset of drug reviews is required. This dataset, called drug review dataset, was collected from *www.druglib.com*, a popular website for reviewing drugs. This dataset contains 280 reviews for 33 drugs which were chosen randomly from the list of the most frequently rated drugs at the first page of the *druglib*.*com* website. We also collected a different dataset of 200 drug reviews from *www.askapatient.com* and used it as development set. The development set in which each indirect opinion is tagged by affected entities is used for determining affected entity types (section “Named entity recognition”).

In addition, the proposed method for resource construction is based on domain knowledge. To obtain domain knowledge, we used *www.dailymed.com* and *www.webmd.com* websites. These websites contain good information about usage and side effects of a drug. We integrated appropriate information of these resources and constructed a knowledge base of known drug effects and side effects, called DomainKB.

### The Proposed Method for Resource Construction

For building the essential resources for indirect opinion mining, we extracted two categories of indirect opinions: complete and incomplete. We defined complete indirect opinion as an opinion with both effective and affected entities. Consider the following example:

e.3) “Accutane cleared my skin.”

The above sentence is a complete opinion in which “Accutane” and “skin” are effective and affected entities, respectively. However, since users of most review sites such as drug review sites write about a single entity in each post, they do not mention the entity’s name in the majority of sentences. Thus, a considerable number of sentences have a missing entity. These sentences are called incomplete indirect opinions. Consider the following example:

e.4) “Helped with sleep”

This example is part of a review about “Strattera” in which the drug’s name has not been mentioned.

**Fig 1:**
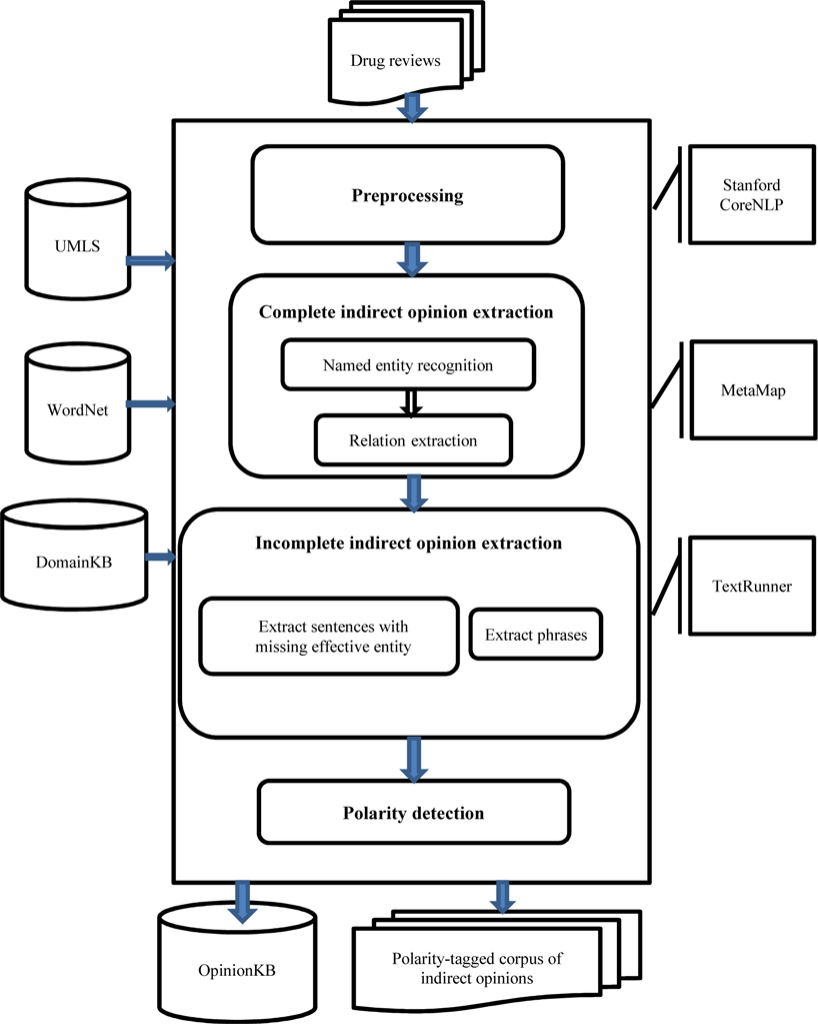
The overview of our method

An overview of our method for resource construction is depicted in Fig 1. As can be seen from Fig 1, the proposed method is composed of four modules: (1) preprocessing, (2) complete indirect opinion extraction, (3) incomplete indirect opinion extraction, and (4) polarity detection. The output is a polarity-tagged corpus of sentences which imply indirect opinions and a knowledge base of quadruples (*e*_*i*_, *e*_*j*_, *r*_*ij*_, *p*), called OpinionKB, extracted from those sentences. In the following subsections, we describe each module in detail.

#### Preprocessing

As shown in Fig 1, the first step is preprocessing of input data. For each review, we first used the Stanford CoreNLP (*http://nlp.stanford.edu/software/corenlp.shtml*) to detect sentences. Subsequently, compound sentences were broken down into simple units. Splitting was done by exploiting dependency tree [44] and conjunction structure of the sentence. Then imperative and conditional sentences were discarded. Although some of imperative and conditional sentences induce opinion, but they require different analysis techniques and we ignored them in this research. Then we performed tokenization, lemmatization and POS tagging on each sentence using Stanford CoreNLP tools. Finally, exploiting the Stanford coreference resolution [45], we replaced each resolved pronoun with the origin term that it refers to.

#### Complete Indirect Opinion Extraction

Extraction and analysis of indirect opinions is different from those of direct opinions. Given a pair of entities, indirect opinion analysis intends to determine polarity of the relationship between them. Thus, complete indirect opinion extraction module consists of the following sub-modules:

1. Named entity recognition: The goal of this sub-module is to recognize the interested entities of the given sentence.
2. Relationship extraction: Given two entities, the goal of this sub-module is to find the relationship between them.

*Named Entity Recognition*.

Named entity recognition is the task of identifying and semantically classifying named entities in text into predefined categories. In this paper, we used MetaMap [46] to identify entities as defined in UMLS (*www.nlm.nih.gov/research/umls/*). The UMLS Metathesaurus includes 1.7 million concepts, grouped in more than 130 semantic types. Each semantic type belongs to one of the 15 semantic groups [47]. Table 2 illustrates the semantic types and semantic groups of entities extracted from the sentence “Over the course of three nights, Avelox caused heart palpitations, confusion, and lasting muscle weakness.”. For example, the concept “course” belongs to the semantic type “Temporal Concept” which in turn belongs to the semantic group “Concepts & Ideas”.

**Table 2:** An example of entities extracted by MetaMap from a drug review.

Entity	Semantic type	Semantic group
course	Temporal Concept (tmco)	Concepts & Ideas (CONC)
three	Quantitative Concept (qnco)	Concepts & Ideas (CONC)
nights	Temporal Concept (tmco)	Concepts & Ideas (CONC)
Avelox	Pharmacologic Substance (phsu)	Chemicals & Drugs (CHEM)
caused	Functional Concept (ftcn)	Concepts & Ideas (CONC)
heart	Body Part, Organ, or Organ Component (bpoc)	Anatomy (ANAT)
palpitations	Finding (fndg)	Disorders (DISO)
confusion	Mental or Behavioral Dysfunction (mobd)	Disorders (DISO)
muscle weakness	Sign or Symptom (sosy)	Disorders (DISO)

As can be seen in Table 2, MetaMap extracts biomedical and health-related concepts as well as general concepts like temporal ones (e.g., “course” and “nights”). However, we are only interested in some of them as affected entities (e.g., “palpitations”, “confusion” and “muscle weakness”). To solve this problem, the idea is to select only the important concepts in the drug domain and remove the general ones. To this end, we modified CF-IOF (concept frequency–inverse opinion frequency) [7], a technique that evaluates how important a concept is to a specific context. This modified form is called SGF-IOF (semantic group frequency-inverse opinion frequency) and is calculated as follows:

$$\text{SGF}\_{\text{IOF}}_{\text{sg},\text{d}}=\frac{{\text{n}}_{\text{sg},\text{d}}}{\sum_{\text{k}}{\text{n}}_{\text{k},\text{d}}}\text{log}\underset{\text{k}}{\sum }\alpha .\frac{{\text{n}}_{\text{k}}}{{\text{n}}_{\text{sg}}}$$ (1)

Where n_sg,d_ is the number of occurrences of semantic group *sg* in the subset of opinions tagged as *d*, n_k_ is the total number of semantic group occurrences, and n_sg_ is the number of occurrences of *sg* in the whole set of opinions. α is a constant parameter that shows the relative importance of SGF versus IOF. This parameter does not exist in the CF-IOF formula, but our experiments on the development set showed that by adjusting it we could achieve better results.

In order to use SGF-IOF, first a set of opinions about different topics i.e., drugs, restaurants (*http://www.cs.cmu.edu/~mehrbod/RR/*) and products [8] were collected. *d* is the development set of drug reviews which was described in section “The Exploited Resources”. Then, each occurrence of an entity mention was replaced with its semantic group, which ensured that the name did not have an influence. Finally, exploiting SGF-IOF technique, we filtered out general semantic groups (i.e., semantic groups with low weight). In fact, we ignored each semantic group which its weight was less than a threshold value *θ*. We used learning automata [48] for adjusting the α and *θ* parameters. In this way, α and *θ* have been set to their best values (i.e., 0.4 and 0.084, respectively) with which we gained the best performance in affected entity recognition on the development set. Finally, we selected important semantic groups as affected entity types. We also assumed that the effective entity should be a drug.

Determining a list of affected entity types, we tagged all effective and affected entities of the sentences with appropriate type using MetaMap. Then we selected each sentence with both effective and affected entities as a candidate for complete indirect opinion.

*Relationship Extraction*.

After identifying effective and affected entities, we need to extract the relationship between them. There are many well-known techniques for relation extraction. We used a rule-based method for extracting relationships between the effective and affected entities. To this end, we performed the following steps:

- We identified the main verb(s) of the sentence using the approach proposed in [49]. This approach only considers the verbs that are semantically similar to one of the verbs listed in UMLS. However, we did not impose any constraint on the verb.
- We applied some predefined rules to determine the object and subject of the sentence using the Stanford Parser.
- If the subject and object of the sentence were one of the legal types for effective and affected entities, the quadruple *(effective entity*, *affected entity*, *main-verb*, *null)* would be added to the OpinionKB.

#### Incomplete Indirect Opinion Extraction

As mentioned before, in an incomplete indirect opinion, the effective entity is missed. Consider the following examples:

e.5) “reduced amount of cystic acne”

e.6) “Lips were chapped and nose was dried.”

e.7) “no acne”

These examples are parts of a review about “Accutane” in which the drug’s name has not been mentioned. We distinguished two categories for incomplete indirect opinions: sentences with missing effective entity (e.g., e.5 and e.6), and phrases (e.g., e.7).

*Sentences with Missing Effective Entity*.

Sentences with missing effective entity were grouped into two categories: active sentences with missing subject (e.g., e.5), and passive sentences (e.g., e.6).

**Fig 2:**
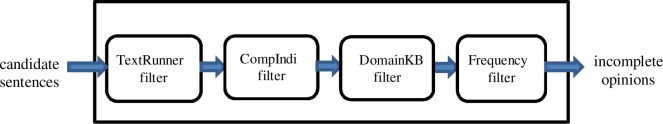
The overall view of the filtering module

At first, we chose each sentence with at least one affected entity and without effective entity as a candidate for incomplete opinion about a drug and extracted the tuple(s) (*v*, *e*), in which *v* was the main verb of the sentence and *e* was the affected entity. However, some of these candidates were noise and had to be filter out. To solve this problem, we proposed a filtering module. An overall view of the filtering module is illustrated in Fig 2.

As shown in Fig 2, the process of filtering unreliable candidates had four main steps. In the first step, TextRunner filter tried to find the missing subject of the input sentence. To do this, it first determined possible candidates for the missing subject, and then it used a score function to judge whether a candidate was credible or not.

In order to determine possible candidates for the missing subject, if the sentence was the first sentence of the review, it would assume that the subject was the drug’s name mentioned in the review’s title. Otherwise, noun phrases of the previous sentences plus the drug’s name would select as candidates for the missing subject. To choose the best candidate, we proposed a method to compute the probability of observing a relation-argument pair in a knowledge base constructed by automatically extracting the relations from webpages.

Given a set of *n* candidates *c*_1_, *c*_2_,…,*c*_*n*_ ∈ *C*, the best candidate was found by querying TextRunner [50]. TextRunner is an open information extraction system, which extracts binary relations from webpages. For each candidate *c*_*i*_, we queried TextRunner in the following way:

$$<A\;rg_{0}\;:c_{i}\;;\;Predicate\;:\;v;\;A\;rg_{1}:e>$$ (2)

where *v* is the main verb and *e* is the affected entity of the sentence. Tuples provided by TextRunner in response to the above query were exploited to measure the probability of observing *c*_*i*_ as the first argument of the relation with the main verb *v* and the second argument *e*:

$$P(<A\;rg_{0}:c_{i}>|<\text{Pr}edicate\;:v\;,A\;rg_{1}:e>)=\frac{\#t<c_{i},v,e>}{\sum_{k}\#t<c_{k},v,e>}$$ (3)

where #*t*〈*c*_*i*_, *v*, *e*〉 is the number of tuples extracted in response to the query < *A rg*_0_ :*c*_*i*_ ; *Predicate* :*v* ;*A rg*_1_ :*e* > and $\sum_{k}\#t<c_{k},v,e>$ is the total number of tuples extracted for all candidates.

Finally, we selected the highest probability candidate as the best candidate in the following way:

$$argmax_{c_{i}\in C}\;P(\langle A\;rg_{0}\;:c_{i}\rangle |\langle Predicate\;:v\;,\;A\;rg_{1}\;:e\rangle )$$ (4)

If the best candidate was an entity of the effective type (drug), we would select the sentence as indirect opinion. Otherwise, it would discard. Consider the following example:

e.8) “My doctor prescribed me Accutane. Within a month, my skin was almost completely cleared.”

In this example, there is an incomplete indirect opinion: “Within a month, my skin was almost completely cleared.”. This sentence has an affected entity, “skin”, but the effective entity is missed. Exploiting the mentioned method, we have two candidates for the missing effective entity: “doctor” and “Accutane”. Querying TextRunner for < *doctor*, *clear*, *skin* >, < *Accutane*, *clear*, *skin* > and <?, *clear*, *skin* >, we reach the following probabilities:

$$\begin{array}{l} P(<A\;rg_{0}\;:A\;ccu\;\text{tan}e>|<Predicate\;:\;clear\;,A\;rg_{1}\;:skin>)=0.041\\ P(<A\;rg_{0}\;:doctor\;>|<Predicate\;:\;clear\;,A\;rg_{1}\;:skin>)=0 \end{array}$$

Hence, “Accutane” is selected as missing effective entity, and the above sentence is chosen as incomplete indirect opinion.

Sometimes, TextRunner did not extract any tuple for none of the candidates. In these cases, we used CompIndi filter. This filter looked for the relation < *drug*, *v*, *e* > in the list of complete indirect opinions extracted in the previous phase. If the relation existed, the sentence would be selected as an incomplete indirect opinion.

In the third step, we used the DomainKB filter. This filter searched the extracted tuple in the DomainKB. If this search led to a result, the tuple would be selected as an incomplete indirect opinion.

Finally, for the remained sentences, frequency filter was used. This filter searched the list of all tuples *(v*, *affected-entity-semantic-type)* extracted from incomplete opinion candidates and chose frequent tuples. In this work, we would define a tuple as frequent if it appeared more than three times. Infrequent tuples were discarded.

After finding the missing effective entity, we created a tuple *(effective entity*, *affected entity*, *main-verb*, *null)* for each opinion and added it to the OpinionKB.

*Phrases*.

Sometimes, authors do not write a complete sentence. Instead, they describe drug’s effects with phrases, which are usually separated by comma. Consider the following examples:

e.9) “Very very dry skin”

e.10) “Extensive dryness on the lips and skin”

e.11) “Dryness of the skin and mouth, peeling/flaking”

e.12) “Clearing of acne”

For phrases, we performed the following steps:

- We determined the adjectives or noun phrases that described the affected entity using some linguistic patterns such as ADJ+NOUN and NOUN+NOUN.
- We created a tuple *(descriptor*, *affected-entity-semantic-type)*. For example, in e.9, the tuple *(dry*, *bdsu)* was created, in which “bdsu” is the semantic type of the affected entity “skin”.
- We searched the extracted tuple in the DomainKB in which each concept was tagged by its semantic type.
- If the tuple existed in the DomainKB, we would select the phrase as incomplete indirect opinion and add the tuple *(drug-name*, *descriptor affected-entity*, *cause*, *null)* to the OpinionKB.
- Otherwise, we created a second candidate list containing tuples *(descriptor*, *affected-entity-semantic-type)* in which the affected entity semantic type or its descriptor did not exist in the DomainKB. A tuple of this list would be an incomplete indirect opinion if the frequency of its occurrences was more than *β* (a constant parameter, called minimum support). Adjusting *β*, we can reach a trade-off between the coverage and accuracy of the knowledge base. The higher value *β* gains, the more accuracy and the less coverage the knowledge base would have. Here, *β* has been set to 3.
- Finally, we created a quadruple *(drug-name*, *descriptor affected-entity*, *cause*, *null)* for each selected tuple of the second candidate list and added it to the OpinionKB.

#### Indirect Opinion Polarity Detection

**Fig 3:**
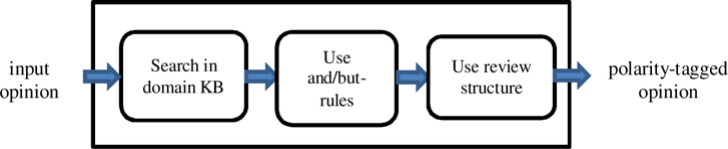
The overall view of the polarity detection module

This module aims to determine the polarity of an indirect opinion. The overall view of this module is depicted in Fig 3.

As can be seen from Fig 3, in the first step the opinion was searched in the DomainKB. If the opinion expressed a known effect (or side effect) of the drug, according to the DomainKB, it would be classified as positive (or negative). In this way, some of the extracted opinions got polarity tag.

The polarity of a quadruple could be affected by a set of valence shifters. In this research, we considered two types of valence shifters which occur frequently in drug reviews: negations and quantifiers. Negation words such as “not” and “no” can change the polarity of an opinion. For example, although “clear skin” is a known effect of “Accutane”, but the negation word “not” implies that the sentence “Accutane did not clear my skin.”, has negative polarity. Likewise, quantifiers which express a decreased/increased value of quantity can change the polarity. For example, the phrase “less acne” has positive polarity, although the word “acne” is negative. To overcome this issue, we applied valence shifter rules described in [5].

In the second step, we applied the following rules as in [5]:

- “and”-rule: Sentences and clauses that are connected with “and”-like conjunctives often have the same polarity. For example, “My skin dried up and the acne went away.” implies that these two sentences have the same polarity.
- “but”-rule: Sentences and clauses that are linked by “but”-like conjunctives often have the opposite polarity. For example, “Since then my skin has been pretty clear, but I still have occasional breakouts.” indicates that the two sentences have opposite polarity.

Exploiting the above rules, if we knew the polarity of one sentence (some sentences got polarity tag in the previous step), we would determine the polarity of another one.

**Fig 4:**
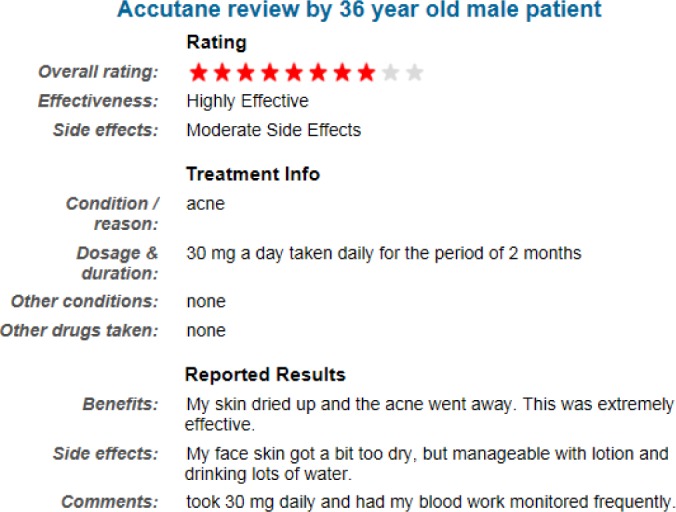
An example of a structured review from www.druglib.com

In the third step, we used review structure to determine the polarity of sentences that remained untagged after two previous steps. In most of review sites, a review is associated with metadata which can be used to determine its polarity. For example, *Druglib*.*com* website asks the reviewers to describe Pros and Cons separately. In fact, the reviewers comment on the benefits and side effects of a drug separately (see Fig 4).

If the review site does not contain the Pros and Cons, we can use the overall opinion rating, which is given by the author to the review. The intuition is that if the overall rating is high, it is very unlikely that there are many negative sentences in the review. Therefore, we can assume that sentences of a high rate review are positive and vice versa. Review sites which have neither Pros and Cons nor overall rating are outside the scope of this research.

To determine the polarity of indirect opinions, we used structure of the review site (i.e., “benefits” and “side effects” fields of a review in *Druglib*.*com*). We defined a basic rule, which tags each sentence in the “benefit” field as positive, and each sentence in the “side effects” field as negative. However, this rule is not always true. Consider the following text written in the “benefits” field of a review:

e.13) “Benefits: Imitrex just isn't right for me. I experienced flushing, sensations of tingling/prickling, weakness, drowsiness, dizziness, fast/pounding heartbeat. On top of that the packaging is just HORRIBLE. It comes 9 to a package. The packaging is a tri-fold booklet that is very rigid and has individual blisters for each tablet. It's about 6 1/2 by 3 1/2 inches, folded. I like to have migraine meds on me at all times so I ended up cutting the tablets out of the booklet so they would fit in my wallet or purse. Definitely going back to Axert!”

The above text was written in the “benefits” field, but the majority of its sentences are negative. Therefore, we assigned a confidence degree to the polarity of an indirect opinion chosen from (0,1]. If the indirect opinion talked about a known effect or side effect of a drug (according to the DomainKB), the confidence degree would be set to 1. Otherwise, the confidence degree would be obtained from the following equation:

$$CD_{\left(o\;,p\right)}=\frac{C^{\;p}}{C}$$ (5)

where *C* ^*p*^ is the number of occurrences of indirect opinion *o* with polarity *p*, and *C* is the total number of occurrences of indirect opinion *o*. Then polarity of the opinion was set to the value with higher confidence degree. For example, if an indirect opinion (*e*_*i*_, *e*_*j*_, *r*_*ij*_, *p*) occurs in the “benefits” fields of drug reviews three times and in the “side effects” fields one time, *p* is set to “positive”.

We only calculated the confidence degree for each opinion, which occurred at least three times. Finally, if we had opinions with unknown polarity, we would determine the polarity according to the basic rule.

#### Developing an Annotated Corpus

Each sentence or phrase which was tagged as indirect opinion in the previous steps was annotated by appropriated tags and then added to the corpus. For each indirect opinion, we tagged its polarity (positive/negative), type of opinion (complete/incomplete), effective-entity and its semantic type and group, affected-entity and its semantic type and group, main-verb and valence shifters. Here are some examples:

e.14) “Advil disappeared my pain.”

annotated text: “[Advil/effective-entity/phsu/CHEM] [disappeared/main-verb] my [pain/affected-entity/sosy/DISO]”

type: complete

polarity: positive

e.15) “No skin irritation”

annotated text: [No/negation] [skin irritation/affected-entity/sosy/DISO]

type: incomplete

polarity: positive

### The proposed Method for Exploiting the OpinionKB

In this section, we propose a method for employing the OpinionKB to detect the polarity of new examples. A simple method is to extract the opinion quadruple from the input example and search it in the OpinionKB. If the OpinionKB involves the tuple, the corresponding polarity tag will be returned. The issue of this method is that many of new examples cannot be classified. In order to overcome the missing knowledge issue, i.e., not all indirect opinions are contained in the OpinionKB, we proposed a pattern-based approach. We defined two categories of patterns: semantic type and semantic group patterns.

To extract semantic type patterns, for each tuple in the OpinionKB, we replaced effective and affected entities with their semantic types. Then we found frequent patterns, i.e., those patterns that were occurred more than a predefined threshold. This threshold was defined by using a trial-and-error approach and was set to 3.

To extract semantic group patterns, for each tuple in the semantic type patterns, we replaced semantic types of the entities with their semantic groups. Then, we found frequent patterns.

Finally, polarity detection was preformed through the following three steps.

- For the extracted tuple of the input example, the OpinionKB was searched. If the knowledge base involved the tuple, the corresponding polarity tag would be returned.
- The entities of the input tuple were replaced with their semantic types. This tuple was searched in the semantic type patterns. If this search led to a result, the polarity tag would be returned.
- Finally, the entities of the input tuple were replaced with their semantic groups. If the resulted tuple existed in the semantic group patterns, the polarity tag would be returned.

## Experiments and Results

Exploiting the proposed method on the drug review dataset (described in section “The Exploited Resources”), a corpus of 936 polarity-tagged indirect opinions and a knowledge base of 896 quadruples were extracted (redundant quadruples were discarded). In this section, we present and discuss the experiments that we conducted to evaluate the quality of these resources.

To evaluate the performance of the proposed method, we used three measures: precision, recall and F-measure. Precision is the percentage of classified instances that are correct, recall is the percentage of instances that are correctly classified, and F-measure is the harmonic tradeoff between precision and recall. The precision, recall and F-measure are defined as follows:

$$\begin{array}{l} \text{precision}=\frac{\text{No}.\;\text{of correctly classified instances}}{\text{Total No}.\;\text{of classified instances}}\\ \text{recall=}\frac{\text{No}.\;\text{of correctly classified instances}}{\text{Total No}.\;\text{of instances}}\\ \text{F}-\text{measure}=\frac{2.\text{precision}.\text{recall}}{\text{precision}+\text{recall}} \end{array}$$ (6)

### Corpus Evaluation

In order to evaluate the quality of the constructed corpus, a subset of the drug review dataset containing 82 reviews for 13 drugs was selected. Then two annotators were asked to identify indirect opinions of the selected drug reviews and annotate them with polarity tags (positive/negative). We only chose those sentences and phrases, which were tagged as indirect opinion by both annotators. In this way, from 82 reviews including 574 sentences and phrases, a collection of 307 indirect opinions was created, of which 46.91 percent were labeled as positive and the rest were negative (53.09 percent). Table 3 illustrates the statistics of the manually created corpus. We assessed our automatically constructed corpus against this manually created corpus.

**Table 3:** Statistics of the manually created corpus

**No. of reviews**	82
**No. of sentences and phrases**	574
**No. of complete opinions**	21
**No. of incomplete opinions**	286
**No. of positive opinions**	144
**No. of negative opinions**	163
**Total No. of opinions**	307

We evaluated the proposed algorithm for extraction of the indirect opinions (and not their polarity) from two aspects: the performance of each module, and the overall performance (Table 4).

**Table 4:** Performance of the indirect opinion extraction modules

Module	Recall (%)	Precision (%)	F-measure (%)
**Complete indirect opinion extraction**	85.71	90.00	87.80
**Incomplete indirect opinion extraction**	86.36	93.20	89.65
**Overall (indirect opinion extraction)**	86.32	92.98	89.53

There are two notable results in Table 4. First, the F-measure of the incomplete indirect opinion extraction module is higher than that of the complete indirect opinion extraction module. That is expected since the complete indirect opinion extraction module needs relation extraction which is a challenging task. In contrast, most of opinions extracted by the incomplete indirect opinion extraction module are simple phrases which are often found in the domain knowledge base. Second, in all cases, the precision is higher than the recall. In our research, precision is more important than recall because the better the precision the cleaner the corpus.

**Fig 5:**
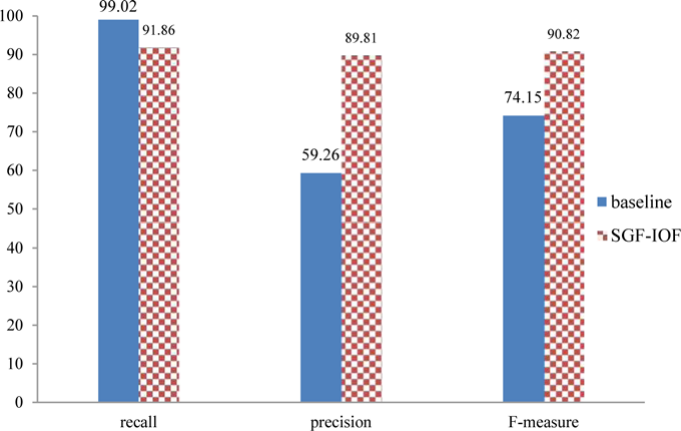
Comparison of the proposed method for detecting affected entities with the baseline method

Fig 5 illustrates the performance of the proposed method for detecting affected entities and compares it with the baseline method performance. In the baseline method, each noun phrase that is tagged by MetaMap as an entity is considered as a candidate for the affected entity of the given sentence. As can be seen from Fig 5, the SGF-IOF technique achieves good performance in comparison with the baseline method. As mentioned earlier, the main reason is that MetaMap tags some kinds of the entities such as temporal ones that are not actually affected entities.

**Fig 6:**
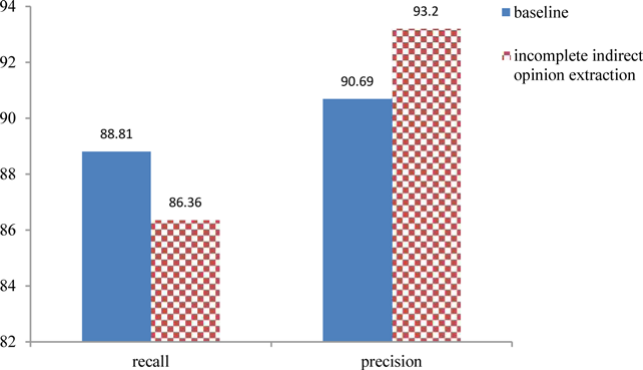
Comparison of the proposed method for incomplete indirect opinion extraction with the baseline method

We also compared the performance of the proposed method for incomplete indirect opinion extraction to a baseline method, where each appearance of an affected entity in a sentence denotes an indirect opinion. In fact, the baseline method is the proposed method for incomplete indirect opinion extraction without the filtering module. Fig 6 shows that the proposed method outperforms the baseline method in terms of precision. As mentioned earlier, since we aim to construct an accurate corpus, precision is more important than recall.

In order to show the effectiveness of the four filtering sub-modules proposed in this research (see Fig 2), we computed the percentage of candidates filtered out by each sub-module and corresponding accuracy. The accuracy is defined as the ratio of number of correctly selected candidates to the total number of candidates. Table 5 compares the participation of each filtering sub-module, in overall accuracy of the filtering module. As can be seen from Table 5, approximately 53 percent of candidates were selected by using TextRunner filter with 96 percent accuracy. CompIndi and DomainKB filters chose about 12 and 28 percent of candidates with 95.65 and 88.46 percent accuracy, respectively. About 7 percent of candidates were selected by frequency filter with 84.62 percent accuracy.

**Table 5:** Performance of the filtering sub-modules

Filtering sub-module	Selected candidates (%)	Accuracy (%)
**TextRunner filter**	53.19	96.00
**CompIndi filter**	12.23	95.65
**DomainKB filter**	27.67	88.86
**Frequency filter**	6.91	84.62

In the next experiment, we evaluated the performance of the polarity detection module. Table 6 presents the performance of the polarity detection module and compares it with a baseline method in which only the basic rule (described in section “Indirect Opinion Polarity Detection”) is used to tag the opinions’ polarity. As can be seen from Table 6, the proposed approach for polarity detection has a significant improvement over the baseline method.

**Table 6:** Performance of the polarity detection module, and comparison with the performance of the baseline method

Polarity detection	Precision (%)
Baseline method	87.61
Our proposed approach	94.34

Table 7 compares the participation of the each sub-module of the polarity detection module (Fig 3), in overall accuracy. As can be seen from Table 7, approximately 31 percent of sentences and phrases were tagged by using domain knowledge with 100 percent accuracy. The “and”/”but” rules assigned polarity tags to 8 percent of opinions and achieved 94.74 percent accuracy. About 61 percent of sentences and phrases were tagged by review structure with 90.91 percent accuracy.

**Table 7:** The accuracy of the polarity detection sub-modules

Polarity detection sub-module	Tagged sentences and phrases (%)	Accuracy (%)
**Domain KB**	31	100
**“and”/”but” rules**	8	94.74
**Review structure**	61	90.91

Table 8 depicts the precision of the indirect opinion extraction, polarity detection and overall system. The overall precision of the proposed approach is 88.42 percent.

**Table 8:** Precision of the proposed system

	Precision (%)
**Indirect opinion extraction**	92.98
**Polarity detection**	94.34
**Overall**	88.42

The preliminary evaluation shows that the proposed approach is able to construct the corpus with acceptable accuracy. However, to perform better evaluation, we need to exploit the constructed corpus to the task of polarity detection. In the future work, we will use this corpus for supervised indirect opinion mining

### Knowledge Base Evaluation

Applying the proposed approach on the drug review dataset, all quadruples (*e*_*i*_, *e*_*j*_, *r*_*ij*_, *p*) were extracted. Some examples of the extracted quadruples are illustrated in Table 9.

**Table 9:** Examples of the extracted indirect opinion tuples

Indirect opinion tuples
*(Accutane*, *dry lip*, *cause*, *negative)*
*(Cipro*, *insomnia*, *cause*, *negative)*
*(Avita*, *acne*, *decrease*, *positive)*
*(Oracea*, *yeast-infection*, *rid*, *positive)*
*(Avita*, *redness*, *cause*, *negative)*

To evaluate the quality of the OpinionKB, we chose a subset of extracted quadruples (i.e., quadruples extracted from the subset of the drug review dataset which labeled by human annotators). We manually checked up these quadruples, and reported the precision as shown in Table 10. As can be seen, the results obtained using the proposed approach are promising.

**Table 10:** Precision of the OpinionKB

No. of extracted quadruples	No. of correctlyextracted quadruples	Precision (%)
302	266	88.08

We also evaluated the OpinionKB by employing it to detect the polarity of new examples. At first, we gathered a test set of 80 examples from the *www.askapatient.com* website. Table 11 shows the properties of the test set.

**Table 11:** Test set information

**No. of sentences and phrases**	80
**No. of complete indirect opinions**	29
**No. of incomplete indirect opinions**	51
**No. of positive opinions**	40
**No. of negative opinions**	40

For each sentence in the test set, the quadruples were extracted following the same process we used for building the knowledge base, with the exception that the filtering phase of incomplete indirect opinion extraction was ignored. In fact, we assumed that each sentence of the test set was an indirect opinion, and therefore, the filtering phase was not needed.

We then performed the proposed method for employing the OpinionKB (see section “The proposed Method for Exploiting the OpinionKB”) to the polarity detection of test examples. Table 12 reports the performance of the proposed method for polarity detection. The first step of this method led to a result in the case of 11 examples. The second step (i.e., using semantic type patterns), tagged 24 examples and finally, the third step (i.e., using semantic group patterns) detected the polarity of 26 examples. For 19 examples, the knowledge stored in the OpinionKB was not sufficient and hence these examples remained untagged.

**Table 12:** Results of polarity detection using OpinionKB

No. of tagged examples	No. of correctly tagged examples	Precision (%)	Recall (%)	F-measure (%)
61	52	85.25	65	73.76

Finally, to investigate the effectiveness of the proposed method for polarity detection, we compared it to some baseline methods and some state-of-the-art opinion mining approaches. We employed a lexicon-based method and a distant-supervision approach as baseline methods. For the lexicon-based approach, we chose SentiWordNet that is a well-known and popular resource for opinion mining. In order to calculate the overall polarity of a text, we first extracted the polarity value of each word using SentiWordNet, and then, we aggregated these values using two methods [51]: majority voting that counts the number of positive and negative words of the text and selects the majority number, and sum of predictions in which the text polarity value is computed as the sum of polarity values of its words.

Distant supervision aims at using a large set of weakly labeled data to train a supervised classifier [52]. We used the structure of review sites to obtain such data. We assumed that sentences in the “benefits” field of a review are positive, and sentences in the “side effects” field are negative. We used the obtained dataset and a set of features exploited by Pang, Lee and Vaithyanathan [9], i.e., unigrams (presence of a certain word, frequencies of words), bigrams, POS tags, and adjectives for training the Naïve Bayes (NB) and Maximum Entropy (MaxEnt) classifiers which are widely used in opinion mining. In our experiments, using unigrams alone yielded the best result.

**Fig 7:**
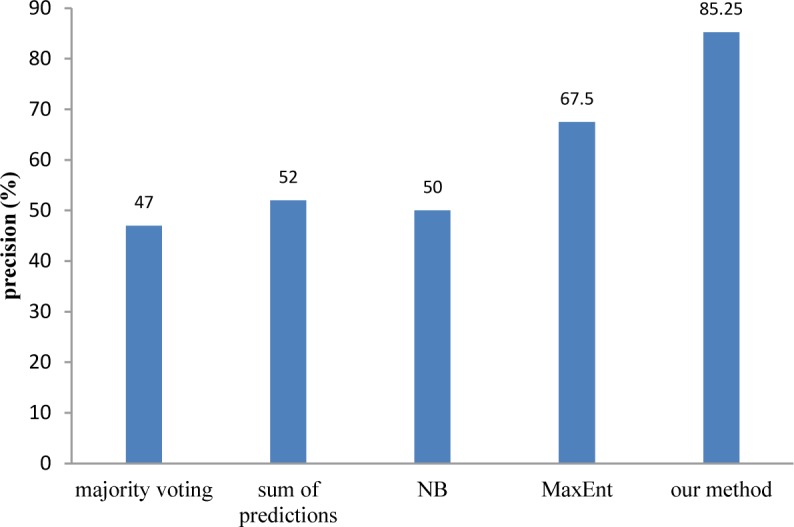
Comparison of the proposed method for polarity detection with the baseline methods

Fig 7 compares the precision of the proposed method for polarity detection with the baseline methods. As can be seen from Fig 7, the proposed method significantly outperforms the baseline methods.

**Fig 8:**
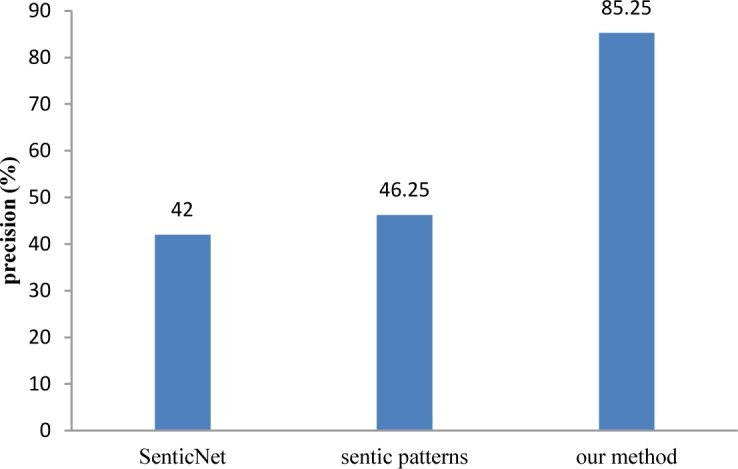
Comparison of the proposed method for polarity detection with other approaches

Fig 8 compares the proposed method with two state-of-the-art approaches for opinion mining. The first approach was proposed by Cambria and Hussain [7]. This approach first deconstructs the given text into concepts using a semantic parser, and then, uses SenticNet, a rich concept-level knowledge base for opinion mining, to associate polarity values to these concepts. Finally, it computes the overall polarity of the text by averaging such values. The second one is sentic patterns [53], a concept-level approach that merges linguistics, common sense computing, and machine learning. To produce results using sentic patterns, we used sentic demo (*sentic*.*net/demo*).

Fig 8 depicts that the proposed method outperforms the state-of-the-art methods. The reported results indicate that current state-of-the-art approaches for direct opinion mining are not appropriate for indirect opinion mining. Therefore, it is necessary to propose new methods for indirect opinion mining.

## Discussion

Despite small quantity of knowledge stored in the OpinionKB, experimental results (Figs 7 and 8) show that it is an appropriate resource for indirect opinion mining. However, there is some room for improving the performance of indirect opinion extraction and polarity detection.

From the error analysis we performed, we have found errors were mainly caused by the following reasons: (1) misspelling words which caused some entities not to be recognized by MetaMap; (2) words and phrases which got wrong tags by MetaMap and caused errors in entity recognition; (3) errors in dependency and parse tree which led to errors in relation extraction between entities; and (4) errors in polarity detection which were mainly caused by the basic rule. These errors indicate how to improve the quality of our proposed approach in the future. To this end, we can use spell checking, alternative tools for parsing or alternative methods for relation extraction. To improve the accuracy of the polarity detection module, we can reduce the effect of the basic rule by extending the domain knowledge base or by proposing some heuristics to correct errors.

## Conclusion and Future Work

Previous approaches for opinion mining have mainly focused on direct opinions. In this paper, we discussed the importance of analyzing indirect opinions, especially in the drug domain. We first formulated the problem of indirect opinion mining. Next, we propose a novel approach for automatic construction of a polarity-tagged corpus and a knowledge base of indirect opinions aiming to provide resources for indirect opinion mining of drug reviews. To this aim, we first presented a model for representing an indirect opinion. Then, based on the proposed model, we designed and populated a knowledge base of indirect opinions. Finally, we proposed a method for polarity detection of new examples using the constructed knowledge base.

Some experiments were designed to evaluate the quality of the constructed resources. The experimental results demonstrated the effectiveness of the constructed resources. We also compared the performance of the proposed method for polarity detection on a dataset of drug reviews to that of some baseline methods and state-of-the-art approaches for opinion mining. The results indicate that the proposed method outperforms these approaches.

As future work, we aim at extending the constructed resources. Exploiting these resources, we also intend to propose a model for indirect opinion mining of unlabeled opinionated texts. In addition, we aim to combine the proposed model with a state-of-the-art approach for direct opinion mining. It is believed that mining indirect opinions besides direct opinions can improve the performance of current opinion mining systems.

## Supporting Information

S1 File Relevant Data. (RAR)
